# Effect of Fiber Post-Resin Matrix Composition on Bond Strength of Post-Cement Interface

**DOI:** 10.1155/2018/4751627

**Published:** 2018-12-02

**Authors:** Ibtisam O. M. Alnaqbi, Haitham Elbishari, Emad S. Elsubeihi

**Affiliations:** ^1^Specialist in Restorative Dentistry, Khorfakkan Hospital, Ministry of Health, Sharjah, UAE; ^2^Assistant Professor, Department of Restorative Dentistry, College of Dentistry, Ajman University, Ajman, UAE

## Abstract

**Objective:**

To evaluate the influence of 3 different post-resin matrix systems cemented with dual-cure resin cement in simulated root canals made of PMMA acrylic sheet.

**Methods:**

3 types of fiber posts (*n* = 60) with different resin matrixes divided into 3 groups: group 1 cross-linked FRC Postec Plus post (*n* = 20), group 2 cross-linked Rely X post (*n* = 20), and group 3 Interpenetrated IPN Everstick post (*n* = 20). All posts were cemented using Multilink Automix dual-cure cement. Posts were cemented into acrylic blocks in order to purely test the strength of cement-post interface. After one week storage at 37°C, two sections of 1 mm thickness from middle-third were subjected to micro-push-out test at crosshead speed 0.5 mm/min.

**Results:**

The data were analyzed using one-way analysis of variance (ANOVA). The variable fiber post-matrix system was found to significantly affect the push-out bond strength (*p* < 0.001). Group 2 exhibited that the highest mean push-out bond strength was (5.36 + 2.3 MPa), and group 3 showed the lowest mean push-out (0.41 + 0.4 MPa). There was significant difference among the groups regarding the failure mode as chi-square test revealed (*p* < 0.001).

**Conclusion:**

Prefabricated cross-linked posts with epoxy-based matrix demonstrated higher bond strength than prefabricated cross-linked posts with Bis-GMA-based matrix and posts with semi-IPN matrix when luted with dimethacrylate-based dual-cured resin cement.

## 1. Introduction

Fiber-reinforced posts have been introduced in the early 1990s [1], as an alternative to prefabricated metal posts to restore endodontically treated teeth with excessive loss of tooth structure. Clinical studies have shown that the most frequent types of failure seen in endodontically treated teeth restored with fiber-reinforced posts were debonding and loss of retention as well as post fracture, [2–7]. Retention of adhesively luted fiber-reinforced posts relies on the strength of the bond between dentine-cement interface on one hand and that of the post-cement interface on the other. It is important that the bond strength of both interfaces is sufficiently strong to withstand stresses during functional loading. Several studies have investigated the bond strength of dentine-cement interface [8, 9]; however, much less attention has been given in investigating the bond strength of post-cement interface based on models that exclude the combined “sandwich” dentine-cement-post assembly [10–12].

In general, fiber-reinforced posts consist of prestretched fibers embedded in a polymer resin matrix. The functions of the matrix in fiber-reinforced posts is to hold the fibers together in the post, as well as interact with functional monomers contained in the adhesive materials for successful bonding of the post to cement resin and composite core materials [13]. Fibers, on the other hand, provide strength and stiffness to the post.

Several manufacturers employ epoxy resin as a matrix for fiber-reinforced posts. Posts with the epoxy resin matrix, however, suffered from poor chemical affinity toward the luting resin because of differences in chemical composition [14, 15]. The introduction of posts with dimethacrylate resin matrix including bisphenol A-glycidyl methacrylate (Bis-GMA) was seen as an advantage toward improving the chemical bonding between the post matrix and that of the resin cement [16]. Studies, however, have shown that the polymer matrix of such fiber-reinforced posts was virtually nonreactive, because the resin has a high degree of conversion and is highly cross linked [16]. This has prompted researchers to explore the options of improving the post-cement bonding through pretreatment of post surfaces with silane either alone [8, 9, 17, 18] or following surfaces conditioning using chemical [9, 14, 18, 19] or mechanical [20] means with varying degrees of success.

The introduction of posts with unidirectional continuous glass fibers embedded in unpolymerized semi-interpenetrating polymer network (IPN) composed of polymethylmethacrylate (PMMA) and Bis-GMA has renewed interests in achieving a true chemical bonding between the post and luting cements. Studies have shown that adhesive systems with monomers that have solubility parameters close to that of PMMA such as Bis-GMA, 2-hydroxyethyl methacrylate (HEMA), and triethylene glycol dimethacrylate (TEGDMA) can penetrate into the linear polymer phase of posts with an IPN matrix but not into posts with an epoxy matrix [21]. The aim of this study is to evaluate the bond strength and mode of failure of fiber-reinforced posts with different resin matrices. The null hypothesis is that the micro-push-out bond strength and mode of failure do not vary with the type of the post-resin matrix when luted with Bis-GMA-based resin cement.

## 2. Materials and Methods

### 2.1. Study Design and Selected Materials

Three types of fiber-reinforced posts with different matrices, namely, (1) cross-linked prefabricated Bis-GMA (FRC Postec Plus, Ivoclar Vivadent, Schaan, Liechtenstein); (2) cross-linked prefabricated epoxy (Rely X Fiber Post, 3M ESPE, Seefeld, Germany); and (3) semi-interpenetrating polymer network of PMMA and Bis-GMA (GC Everstick, StickTech LTD, Turku, Finland) was tested. All posts were luted using Bis-GMA-based, dimethacrylate dual-cured cement (Multilink Automix, Ivoclar Vivadent, Schaan, Liechten) in simulated root canals made of PMMA (Yearlong Industrial Company Limited, Taiwan) with a custom-prepared post space. Composition of posts and luting cement used in this study is illustrated in Table 1.

**Figure 1:**
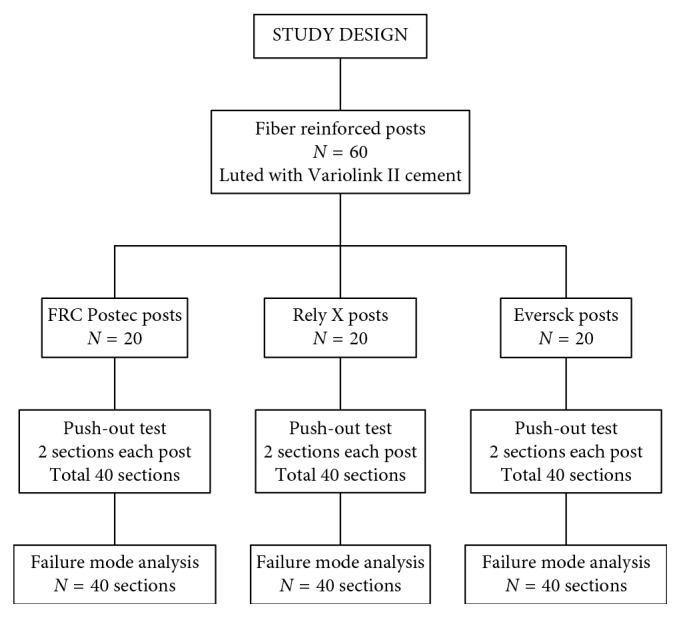
Study design. Figure shows the groups, number of posts in each group, and number of sections analyzed. *N* = sample size.

The push-out bond strength of twenty samples of each cemented post on two sections made from each post was assessed. Furthermore, the mode of failure of all sections following push-out bond strength test was evaluated. Study design is demonstrated in Figure 1.

### 2.2. Fabrication of the Experimental Model

**Figure 2:**
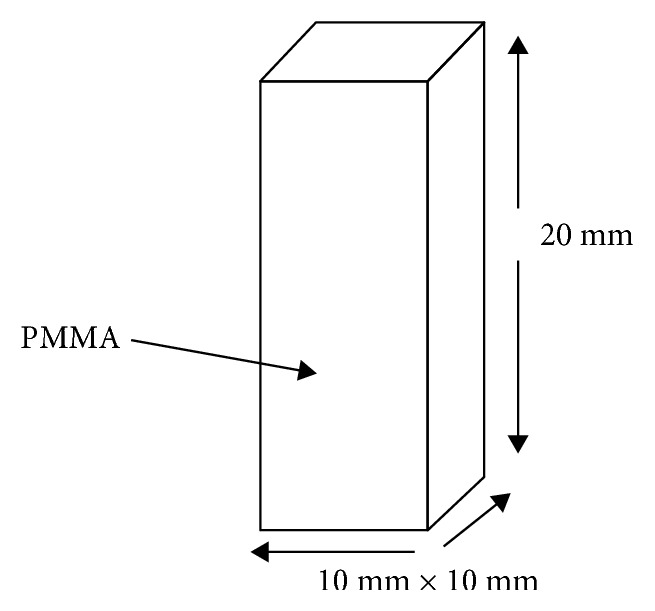
Schematic drawing represents shape and dimension of experimental acrylic block, which is made of PMMA (polymethylmethacrylate). Blocks were cubical in shape with 20.0 mm height and 10.0 mm × 10.0 mm base.

PMMA acrylic sheet (Yearlong Industrial Company Limited, Taiwan) was used to make 60 blocks. The blocks made were cubical in shape with 20 mm height and 10 mm × 10 mm base (Figure 2). The blocks were prepared by cutting the sheet with laser-guided machine (Source Company, Ajman) with high speed, good cooling, and low cutting force as recommended by the manufacturer. Each block was then polished using a polishing machine (MetaServ 250 twin Grinder-Polisher, Buehler, USA) with speed 70 rpm to correct any deviation in the blocks dimension while preparing the blocks.

### 2.3. Post Space Preparation and Cementation

Space for fiber-reinforced post was prepared in each block with size 1 FRC Postec Plus Reamer (Ivoclar Vivadent, Schaan, Liechtenstein) in a low-speed hand piece mounted on a parallel milling device (Amann Girrbach, Vorarlberg, Germany). At the beginning, each block was placed in the block holder perpendicular to the drilling bur at a 90^o^ angle. The blocks were then drilled with intermittent light force at 18,000 rpm speed to the length of 12 mm depth for FRC Postec Plus and Rely X fiber post and 10 mm for the Everstick post. Cooling with normal saline was used to reduce heat generation during the drilling procedure. Post space was then roughened using the Hedstrom endodontic file size 45 (Technical and General Ltd, London, UK) to increase the bond strength between the acrylic block and the cement. The post space was irrigated with alcohol to ensure no grease or debris in the post space walls then dried with paper points. This was followed by irrigation with distilled water and dried using paper point. Following the cleaning and drying stage, the patency of the post space was confirmed by trying in different types of posts to ensure that each type of the post can be seated to the full-prepared post space length.

Each block was painted with black color using a paint pen marker in order to prevent light penetration during and after cementation. Multilink Automix, dual-cure luting cement (Ivoclar Vivadent, Schaan, Liechten) was used to cement all posts according to manufacturer's instruction.

The sample was then placed in a 16.0 mm deep custom-made white container with a 4.0 mm deep plastic cover, which was covered with aluminum foil to prevent undesirable exposure to light. A 5 mm opening was made on the cover to allow the curing of the sample. After that, the cement was cured for 60 seconds with LED (light-emitting diode) curing unit at 600 mW/cm^2^ in high intensity mode (Litex 696, Cordless Led Curing Light, DentAmerica, USA). The tip of the curing unit was placed close to the coronal surface of the post according to the manufacturers' instruction. Light intensity was confirmed with a visible curing light meter (Cure Rite, Dentsply Caulk, Milford, USA) before curing each post. After curing, all specimens were stored at 37°C in 100% humidity in an incubator for 1 week.

### 2.4. Sample Preparation

After 1 week of storage time, acrylic blocks were placed in holder of a saw machine (IsoMet 1000 Precision, Buehler, USA) with post long axis perpendicular to the saw blade disc. The samples were cut into 1.0 mm sections at a speed of 350 rpm. Two sections of 1.0 mm thickness representing the middle third of each post were used for the study. The location of the first section was about 5.0 mm from the coronal end of each post. As the thickness of the cutting blade was about 0.5 mm, the second section used for the study was about 6.5 mm from the coronal end of each post.

The coronal surface of each section was marked with a waterproof marker to identify the coronal and apical surfaces of each section. Any sharp edges in the specimen were then removed by low-speed polishing disk, and thickness of all slices was measured by a laser scan micrometer (Laser Scan Micrometer, LSM-6200, Mitutoyo, Japan) and by a digital caliper.

**Figure 3:**
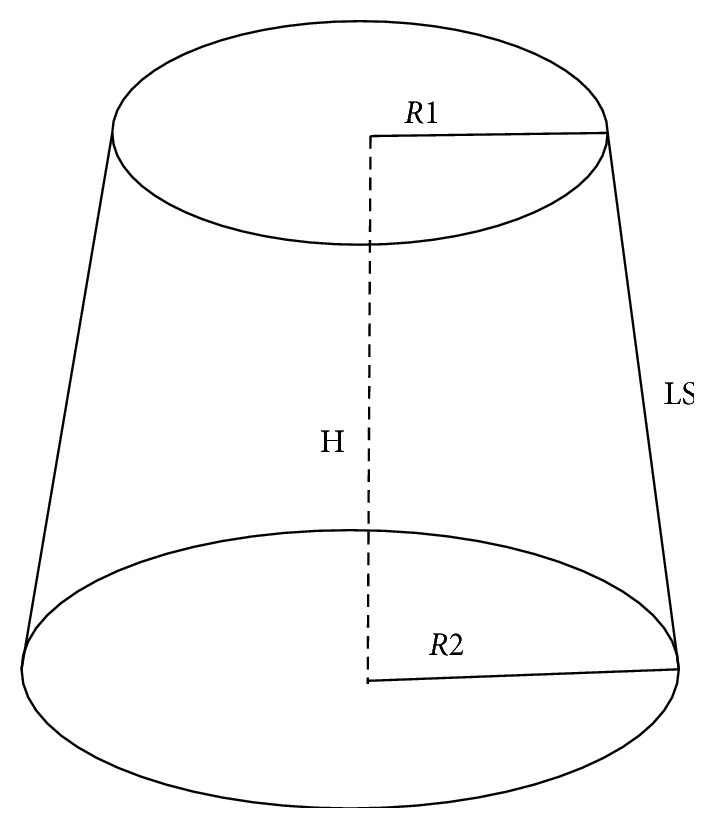
Measurement of the lateral surface area. Schematic drawing shows the post space radius used to calculate the lateral surface area (LS = lateral surface, *R*2 = coronal post space radius, *R*1 = apical post space radius, and *H* = slice thickness). Note that during push-out testing, each section was placed so that the apical side of each section is in direct contact with the push-out pin of the testing machine.

Each section was divided into 4 equal segments using a pencil, and each quarter was color-coded and numbered to be used later for failure mode analysis. Furthermore, the lateral surface area of each post space preparation was calculated. To do this, each section was placed under a stereomicroscope with a mounted camera (EZ4HD, Leica, Singapore) at magnification of × 8, and a photograph was taken. Photographs of both coronal and apical sides of each section were made. Each image was calibrated, and the radius of the coronal and apical sides of each post space was measured using an image analysis system (Image J Software, Java 1.6.0 (64-bits) 1.50i, National Institute of Health, USA). The lateral surface area of each section (Figure 3) was then calculated using the following formula:

(1) $$\begin{array}{c} \text{LS}=\pi \left(R2+R1\right){\left[H^{2}+{\left(R2-R1\right)}^{2}\right]}^{0.5}, \end{array}$$

where LS = lateral surface area, *R*2 = coronal post space radius, *R*1 = apical post space radius, and H = slice thickness.

### 2.5. Measurement of Push-Out Bond Strength

**Figure 4:**
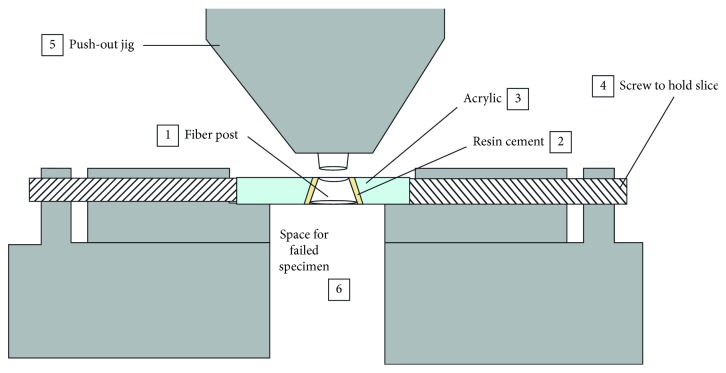
Schematic drawing of the push-out testing set up. The section consisting of (1) fiber post; (2) resin cement, and (3) acrylic block is mounted on the stainless steel base and held in place with screws (4). The push-out pin (5) positioned on the center of the post with a space in the middle of the supporting base to allow collection of the failed post.

The push-out bond strength was measured using a universal testing machine (Universal Testing Machine M350-5CT, Testometric, UK). Each section was placed on a custom-made stainless steel base with the apical side facing upwards under the universal testing machine (Figure 4). A push-out pin of 0.8 mm in diameter was attached to the loading cell of the testing machine. The push-out pin was positioned over the center of the post, so that the force is applied to the post surface without stressing the surrounding post space. A constant load was applied in an apical-coronal direction of each section at a crosshead speed of 0.5 mm/minute. The peak force at the time of post segment extrusion from the section was taken as the point of bond failure, and the value was recorded in Newton (N). Bond strength in megapascal (MPa) was calculated by dividing the force (N) over the lateral surface area (mm^2^) of each section.

### 2.6. Failure Mode Analysis

After push-out testing, all sections were analyzed under a stereomicroscope (EZ4HD, Leica, Singapore) at 35X magnification to determine the mode of failure. As each post acrylic block on each section was labeled with colored markers, it was possible to identify post surface and its corresponding simulated canal surface during assessment of mode of failure. Modes of failure were divided into six types as described in Table 2. As each post acrylic block was divided into 4 segments, it was possible to determine if the resin luting cement covered 50% or more of the surface during assessment of types 5 and 6 mixed failure modes. A single operator determined the failure mode and the coefficient of variation was determined by measuring 30 samples on two different occasions that were three weeks apart.

### 2.7. Statistical Analysis

Statistical analysis was carried out using SPSS software (SPSS version 20, IBM, USA). The push-out bond strength data in MPa were analyzed using one-way analysis of variance (ANOVA). If a significant difference (*p* < 0.05) was found between groups, and the differences were revealed using the Tukey HSD post hoc test. Failure mode analysis was analyzed using the chi-square test. The intraexaminer agreement for mode of failure was assessed using kappa statistics.

## 3. Results

The mean and standard deviations of push-out bond strength (in MPa) of the three types of posts were calculated. Rely X Fiber Post has demonstrated mean bond strength of 5.37 ± 2.30 MPa, whereas FRC Postec Plus post has demonstrated mean bond strength of 2.93 ± 1.88 MPa, and GC Everstick post demonstrated mean bond strength of 0.41 ± 0.41 MPa.

**Figure 5:**
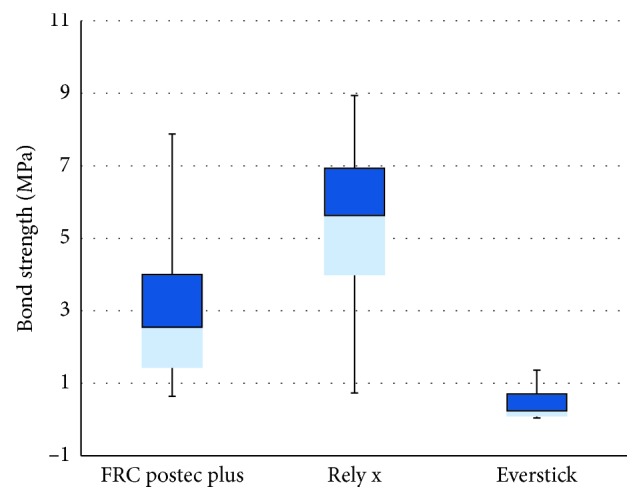
Box plots illustration of push-out bond strength (in MPa) of FRC Postec Plus, Rely X, and Everstick posts.

ANVOA statistics have demonstrated a highly significant difference between groups (*p* < 0.001). The differences between groups were revealed using Tukey HSD. Rely X Fiber post exhibited the highest bond strength, which was significantly higher than both FRC Postec Plus post and GC Everstick post. Furthermore, the bond strength of the FRC Postec Plus post was significantly higher than the bond strength of the GC Everstick post. All push-out bond strength data are illustrated in Figure 5.

Counts and percentages of the different modes of failures are presented in Table 3. It can be seen that no group has demonstrated failure mode type 4 where there was adhesive failure between luting cement and the simulated acrylic canal in this study.

The highest failure mode observed for FRC Postec Plus post was type 6 failure (60%) followed in order by type 5 (25%); type 2 (7.5%); type 3 (5%); and type 1 (2.5%). For the Rely X post, the highest failure mode observed was type 6 (87%), followed in order by type 5 (10%) and type 2 (2.5%). No failure modes of types 1 and 3 were observed for Rely X post. On the contrary, the highest mode of failure observed for the GC Everstick post was type 1 (80%). Furthermore, GC Everstick post demonstrated 7.5% occurrence of types 3 and 6 followed by type 2 (5%). No failure mode type 5 was observed for the GC Everstick post. In all post groups, no failure mode of type 4 was observed.

**Figure 6:**
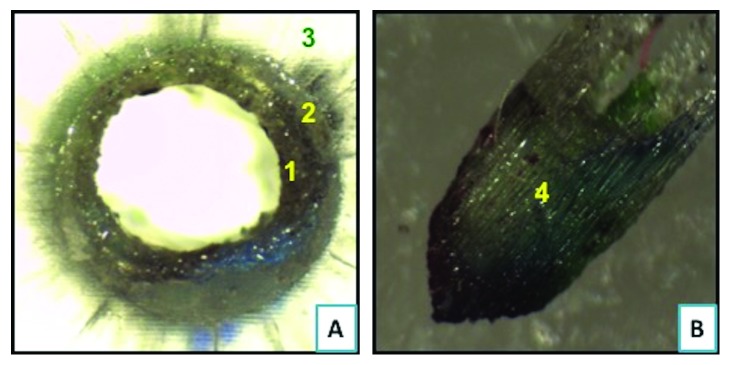
Failure mode type 1 demonstrating cohesive failure within the fiber post. (a) Top view of a section following bond strength testing demonstrating remnants of failed fiber post (1), resin cement (2), and PMMA block (3). (b) A lateral view of the corresponding fractured fiber post following bond strength testing. Pictures were taken at 35X magnifications under stereomicroscope.

**Figure 7:**
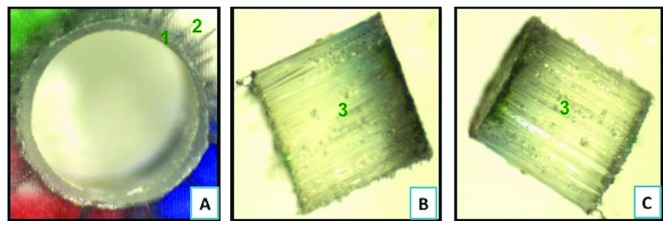
Failure mode type 2 demonstrating adhesive failure between fiber post and the resin cement. (a) Top view of a section following bond strength testing demonstrating resin cement (1) lining the simulated canal made in the PMMA block (2). (b) A lateral view of segments 1 and 2 of the corresponding failed fiber post (3). (c) A lateral view of the same fiber post (3) with no remnants of cement around the fiber post. Pictures were taken under stereomicroscope at 35X magnifications.

**Figure 8:**
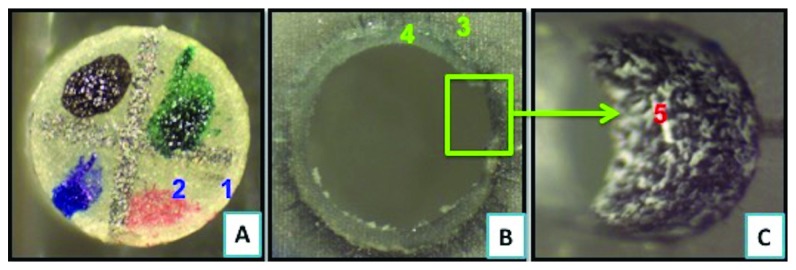
Failure mode type 3 demonstrating cohesive failure within the cement. (a) Top view of the fiber post with the resin cement (1) encircling the whole failed post (2) after bond strength testing. (b) Top view of the simulated acrylic resin canal (3) showing resin cement (4) covering the whole simulated resin canal. (c) Lateral view of inner surface of simulated acrylic resin canal (5). Pictures were taken under stereomicroscope at 35X magnification.

**Figure 9:**
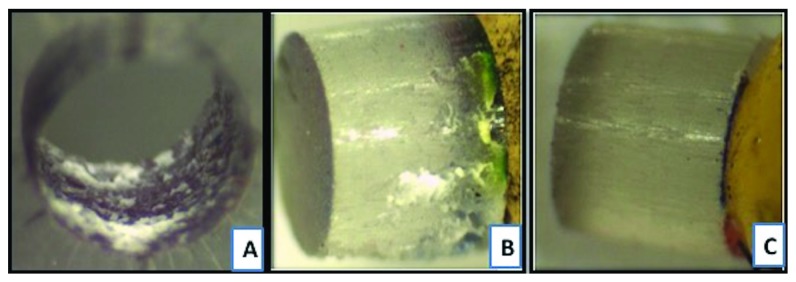
Failure mode type 5 demonstrating a mixed type failure where resin cement (1) covers 25–50% of the post surface (2) following bond strength test. (a) Lateral view of the simulated acrylic resin canal with resin cement (3). (b) Lateral view of the corresponding failed fiber post (4) where cement covers one segment (25%) of the post. (c) Lateral view of the same fiber post where no cement covers the other segments of the post. Pictures were taken under stereomicroscope at 35X magnification.

On comparison of type-2 failure mode, where there is adhesive failure between the cement and the post surface, it is found that FRC Postec Plus sustained the highest failure (7.5%) as compared to GC Everstick (5%), whereas Rely X post sustained the lowest type 2 failure (2.5%). Representative samples of different types of failure as seen under the stereomicroscope are shown in Figures 6–9.

Intraexaminer agreement of assessment of failure mode was evaluated using kappa statistics. The results revealed high intraexaminer agreement (97.3%) for mode of failure (*p*=0.0001).

## 4. Discussion

It is believed that the longevity of the restoration is predicted to some extent by its adhesive ability, which can be measured by bond strength testing [22]. Studies evaluating bond strength are based on the assumption that the higher the bond strength is, the more likely the bonded surfaces survive the functional loads and, therefore, may provide longer survival of the restorations [23, 24]. Although the validity of bond strength tests to predict clinical performance has been questioned [25], existing evidence shows that clinical performance can be predicted by the use of appropriate types of laboratory tests [26, 27]. In addition to bond strength measurements, the observation of failure mode can indicate how the bonding system is working and point out the weakest link [23].

Push-out bond strength test, in this study, revealed that Relay X post had higher bond strength values than FRC Postec Plus and Everstick fiber posts when luted with dual-cured diamethacrylate-based luting agent containing Bis-GMA, HEMA, and UDMA. In agreement with these results, a previous study [10] found that the bond strength of Rely X post was higher than that recorded for FRC Postec Plus using a variety of luting agents when tested using 2.0 mm thick sections made of composite CAD-CAM-simulated canals.

According to the results of our study, Everstick fiber post demonstrated the lowest bond strength values than both Rely X and FRC Postec Plus posts. In accordance with these results, a previous study [28] found that FRC Postec Plus has higher bond strength than Everstick posts when luted with Variolink II cement. The same authors also found another fiber post with epoxy matrix, namely, DT Light fiber posts, to have higher bond strength values than Everstick posts when luted with Variolink II cement [28]. In their study, however, there was no difference in bond strength between DT Light and FRC Postec Plus [28].

Bonding between fiber-reinforced posts and resin cements can occur by different mechanisms including micromechanical interlocking, chemical adhesion, and/or interdiffusion. The polymer matrix of fiber posts with semi-IPN is composed of two independent polymer networks that are not linked by chemical bonds [29]. These consist of a linear polymer phase of PMMA interlaced with a dimethacrylate (Bis-GMA) as a cross-linked phase of the polymer matrix with an enriched layer of PMMA present on the surface of the post [21, 29]. It has been shown that penetration of resin cement into Everstick post with prepolymerized semi-IPN resin matrix was improved by an interdiffusion bonding mechanism [21, 29]. Furthermore, the presence of adhesive resins with solubility parameters close to that of PMMA such as HEMA, Bis-GMA, and TEGDMA are able to penetrate deeper into the IPN polymer structure of Everstick posts [21]. Upon polymerization, a bond based on a secondary semi-IPN structure is formed, which bonds the adhesive cement to the fiber-reinforced post [30].

The ability of HEMA-based resins to penetrate into the polymer of prefabricated cross-linked Bis-GMA-based fiber-reinforced composites has been shown to be significantly lower than that into IPN polymer structure of Everstick C&B fiber-reinforced composites [29]. Although the penetration of these dissolving monomers (HEMA) into cross-linked fiber-reinforced composites was very low, it is unknown if similar penetration of dissolving resins can occur in cross-linked Bis-GMA-based fiber-reinforced posts. On the contrary, studies have shown that monomers such as HEMA, Bis-GMA, and TEGDMA were not able to penetrate the surface of prefabricated cross-linked epoxy-based fiber posts [21]. These findings clearly indicate that interdiffusion does not play any role in adhesion of resin cements to cross-linked epoxy-based fiber-reinforced posts.

It is known that when composites are polymerized in air, a nonpolymerized surface layer, so-called oxygen inhibited layer, will remain on the surface to which resin cements can adhere to by free radical polymerization for chemical bonds [30]. However, the polymer matrix of prefabricated cross-linked posts such as that seen in posts with dimethacrylate-based or epoxy-based matrix is polymerized to a high degree of conversion [31]. This high cross-link density makes it difficult to chemically bond posts with cross-linked dimethacrylate-based or epoxy-based matrix to resin cements [31, 32]. This is due to the fact that the monomers of the luting cements cannot penetrate into the polymer matrix of a cross-linked nature [31–33].

Different prefabricated fiber-reinforced posts have been shown to have different surface topography. A SEM analysis has shown the untreated surface of Rely X posts to have rougher surface than FRC Plus posts [10]. The higher bond strength of Rely X post observed in our study might be, therefore, due to micromechanical interlocking of the resin cement into irregularities at the surface of the post. The notion that micromechanical bonding plays a significant role in bonding of the cross-linked posts tested in our study is further supported by the findings of Le Bell et al., [31] who compared the pull-out force to dislodge smooth- and serrated-surfaces fiber-reinforced posts with IPN polymer matrix and cross-linked epoxy-based matrix to that of serrated titanium posts cemented with resin cement. They found that the highest pull-out force was demonstrated by the titanium serrated posts cemented with resin cement [31]. As real chemical adhesion between prefabricated cross-linked fiber posts with epoxy-based or dimethacrylate-based matrix is unlikely to occur [23], it appears, therefore, that the adhesion between these cross-linked posts used in our study and the dimethacrylate-based cement is mainly mechanical based on the interlocking of the adhesive cement into surface irregularities on the post surfaces [31].

In one study, semi-IPN polymer matrix Everstick post showed higher pull-out bond strength than four types of prefabricated epoxy-based cross-linked fiber-reinforced posts [31]. However, in that study, Everstick posts were light polymerized by exposing all sides of each post to a light-curing unit for 40 seconds each before cementation with self-cured luting cement. Furthermore, all posts were cemented in disks of 2.2 ± 0.1 thick made of composite core material and subjected to macro-pull-out test. In our study, Everstick posts were cured for 60 seconds using an LED light unit after placement in the simulated root canal following the manufacturers' recommendations. Furthermore, neither Rely X nor FRC Postec Plus post was used in the study of Le Bell et al., [31]. In addition, we used the micro-push-out method to test the bond strength, which is regarded as more sensitive in testing bond strength as compared to the macro-push-out testing used in the study of Le Bell et al., [31].

The most common mode of failure observed with GC Everstick posts in our study was cohesive failure within the post (type 1), which occurred in 80% of samples. In addition, one sample of the FRC Postec Plus has demonstrated cohesive failure within the post, whereas no such failure mode was observed in the Rely X posts. The high incidence of cohesive failure within Everstick posts has been observed in other studies. Previous study [23] found that all Everstick posts (100%) tested exhibited cohesive failure within the post. Furthermore, Kececi et al., [28] examined the mode of failure on selected samples and found that 6 out of the 10 Everstick post samples examined exhibited cohesive failure within the post, whereas another 3 samples exhibited mixed adhesive failure at the cement-dentine surface with cohesive failure within the post. Strength and rigidity of fiber-reinforced posts depend on the type of reinforcing fibers and the polymer matrix. Factors such as the type, properties, impregnation, quantity, direction, and density of the reinforcing fibers in addition to the adhesion of the fibers to the matrix and properties of the matrix can influence the mechanical properties of fiber-reinforced posts [30]. While Rely X posts contain S-glass fibers, both Everstick and FRC Postec Plus posts contain E-glass fibers. S-glass fibers are known to have the highest tensile strength among all types of glass fibers, whereas E-glass fibers have lower tensile strength as compared to S-glass fibers [34]. Furthermore, Studies have shown that the intensity of light is significantly reduced from cervical to apical regions of canal space [35, 36] following curing of dual-cured cements because of attenuation of light as it passes from cervical to apical end of the canal [37].

The high incidence of cohesive failure of Everstick posts could be due to the lower mechanical properties of E fibers [34] and/or lower light intensity penetrating into Everstick post within the canal. As Everstick posts were cured after placement in the simulated canal, in this study, the effect of light attenuation on complete curing of Everstick posts cannot be ruled out, because this might have affected the degree of conversion resulting in lower mechanical properties of Everstick posts that can be partially responsible for the high incidence of cohesive failure within the posts seen in this study. The degree of conversion of Everstick posts at different depths of the canal space requires further investigation.

Type 2 cohesive failures between the post surface and the luting cement used in this study were observed in 3 sections of the FRC Postec Plus and 1 section of the Rely X posts. Although no difference in the incidence of cohesive failures between both posts and the luting cement was observed, the bond strength values indicated that stronger adhesion has occurred between Rely X posts and the luting agent than that between FRC Postec Plus and the same luting agent. This is further supported by the observation that 87% of Rely X post samples demonstrated a predominantly adhesive failure (mixed type 6 failure) where 75% of the post surface was covered with the cement as compared to 60% of the samples of FRC Postec Plus posts.

This study investigated the micro-push-out bond strength and failure mode of the post-cement interface by luting posts in customized post space preparation made in PMMA blocks. The use of simulated root canal made in PMMA blocks permitted the evaluation of the post cement interface without interferences from the variables of the cement-dentine interface. When extracted teeth are used for assessment of the post-cement interface, the variables such as the method of dentine preparation, the type of dentine pretreatment, and the type of dentine within the root might affect the bond strength results [10, 38–40]. Furthermore, the lower bond strength values recorded for the cement-dentine interface could have not allowed an accurate evaluation of the bond strength of the cement-post interface [11]. Few other studies investigated the bond strength of the post-cement interface using simulated canals made of composite CAD-CAM blocks [10] or plexiglass root canals [11]. The absence of adhesive failure between cement and PMMA block used in this study suggested that the model used in the current study was reliable in testing the post-cement interface. To our knowledge, the PMMA blocks used in this study have not been used in any previous study to investigate the bond strength of the post-cement interface.

Standardized post space preparations were made using a calibrated drill of FRC Postec Plus® reamer size 1 in a low-speed hand piece while the PMMA blocks were mounted on a parallel milling device to avoid any deviations in post space size. However, post preparation depth of 12.0 mm was made for the FRC Postec Plus and Rely X fiber posts, whereas 10.0 mm post length for the GC Everstick post was made to compensate for the difference in the different post diameters. This assured that the tested sections made 5.0 mm from the coronal end of each post and in the middle of the simulated canals have similar diameters.

Post space was roughed using Hedstrom file to increase the bond strength between the PMMA block and the cement and, therefore, prevent early dislodgment of the cement from the simulated acrylic canals. Other studies [11] have used small round stainless steel bur in order to roughen post space. The advantage of using Hedstrom file was that it might have created less change in the diameter of the simulated canal as compared to round bur in a slow-speed hand piece.

During the cementation procedure, each post was brushed with thin cement layer for 30 seconds before placement in the canal to increase penetration of resin cement to the post surface before curing as has been suggested in previous studies [21, 29].

In this study, all PMMA blocks were painted with black color and placed inside a container that was covered with tin foil during light curing of each post. This is done in order to resemble clinical situation, where light curing is performed through the coronal end of the post, therefore, prevent light exposure to the sides of each post during curing. It has been shown that light penetration depth of each post is different, and this may have an effect on the polymerization of the cement, which may affect the bond strength [41–44]. Other studies investigated post-cement interface in which posts were cemented in simulated canals made in plexiglass blocks [11] or composite CAD-CAM blocks [10], and light curing was performed without blocking the blocks, which could have affected their results.

An LED light curing unit was used to polymerize the resin cement for 60 seconds after placement of each post in its respected simulated canal. The use of the LED curing light has been shown to increase the bond between resin cement and fiber post [45]. Following curing, the samples were stored at 37°C with 100% humidity in the incubator for 1 week before testing. Previous studies have noted the bond strength of adhesively luted fiber posts to be higher when tested 1 week after cementation as compared to those tested 24 to 48 hours after cementation [46–49]. This is possibly because of increased degree of conversion of dual-cured cements over a period of one week [46, 47].

Several studies investigated the bond strength of luted posts in the coronal, middle, and apical thirds of the root canal [10, 50, 51]. However, finite element analysis studies suggested that the highest forces in root canals restored with fiber posts are generated in dentine around the middle third of the canal [24]. This study, therefore, investigated bond strength of two sections of 1.0 mm thick obtained from the middle third of post-simulated canal assembly.

Studies have identified five different modes of failure [17, 28, 52, 53]. In this study, six different modes of failure were defined and identified. Furthermore, in order to differentiate between different mixed types of failure (type 5 and 6 mixed failures), the post-simulated canal-resin block of each post was divided into four equal segments. This allowed better evaluation of the percentage of post surface covered by the cement.

In this study, single operator carried out all failure mode evaluations. Furthermore, the intraexaminer agreement of failure mode was determined by repeated evaluation of 30 samples twice conducted on two different occasions with three weeks apart and found to be 97.3%. While other studies have indicated that one operator conducted failure mode analysis, the coefficient of variation of measurements was not reported in any of the studies [11, 23, 24, 28, 52].

## 5. Conclusions

Within the limitations of this study, it can be concluded that prefabricated cross-linked posts with epoxy-based matrix demonstrated higher bond strength than prefabricated cross-linked posts with Bis-GMA-based matrix and posts with semi-IPN matrix when luted with dimethacrylate-based dual-cured resin cement. Furthermore, posts with different matrices exhibited different failure modes.

## Figures and Tables

**Table 1:** Description of fiber posts and luting cement used in this study.

Fiber post	Manufacturer and batch number used	Matrix	Curing condition of matrix	Fibers	Fillers	Size and shape
GC Everstick	StickTech Ltd., Turku, Finland	Semiinterpenetrating polymer network of PMMA and Bis-GMA	Uncured (to be cured by the clinician)	Unidirectional silane-coated E-glass fibers (61.5% by weight)	No filler	0.9 mm Custom

FRC Postec Plus	Ivoclar Vivadent, Schaan, Liechtenstein	Dimethacrylates (ethoxylated bisphenol A dimethacrylate, Bis-GMA, and 1,4-butanediol dimethacrylate)	Cured by the manufacturers	E-glass fibers	Ytterbium fluoride	0.8–1.5 mm Taper

Rely X Fiber post	3M ESPE, Seefeld, Germany	Epoxy-resin	Cured by the manufacturers	S-glass fibers (60–70% by weight)	Zirconia filler	0.8–1.6 mm Double tapered

Multilink Automix luting cement	Ivoclar Vivadent, Schaan, Liechtenstein	Base		Catalyst		
		(i) Ytterbium trifluoride		(i) Ytterbium trifluoride		
		(ii) Ethoxylated bisphenol A dimethacrylate (Bis-EMA)		(ii) Ethoxylated bisphenol A dimethacrylate (Bis-EMA)		
		(iii) Bisphenol A-glycidyl methacrylate (Bis-GMA)		(iii) urethane dimethacrylate (UDMA)		
		(iv) 2-hydroxyethyl methacrylate (HEMA)		(iv) 2-hydroxyethyl methacrylate (HEMA)		
		(v) 2-Dimethylaminoethyl methacrylate (DMAEMA)		(v) Dibenzoyl peroxide		

**Table 2:** Types of failure mode of different groups investigated.

Type of failure	Description of failure mode
Type 1	Cohesive failure within the post

Type 2	Adhesive failure between post and luting cement with no cement on post surface can be detected. This is considered total adhesive failure between post surface and the luting cement

Type 3	Cohesive failures within the luting cement where the luting cement covers all post surface and the simulated acrylic canal

Type 4	Adhesive failure between the luting cement and the simulated acrylic canal where the luting cement covers all post surface and no luting cement covers the simulated acrylic canal

Type 5	Mixed failure subtype-1 where the luting cement covers 25–50% of the post surface. This is regarded as predominantly adhesive failure between the post surface and the luting cement

Type 6	Mixed failure subtype-2 where the luting cement covers 75% of the post surface. This is regarded as minimal adhesive failure between the post surface and the luting cement

**Table 3:** Counts and % of failure mode among the three post groups tested.

Group	Mode of failure					
	Type 1	Type 2	Type 3	Type 4	Type 5	Type 6
FRC Postec Plus	1 (2.5%)	3 (7.5%)	2 (5.0%)	0 (0.0%)	10 (25%)	24 (60%)
Rely X	0 (0.0%)	1 (2.5%)	0 (0.0%)	0 (0.0%)	4 (10%)	35 (87%)
Everstick	32 (80%)	2 (5.0%)	3 (7.5%)	0 (0.0%)	0 (0.0%)	3 (7.5%)

## Data Availability

The push-out bond strength and failure mode data used to support the findings of this study are available from the corresponding author upon request.
